# *N*′-(2-Bromo­benzyl­idene)-3,4,5-tri­methoxy­benzohydrazide methanol solvate

**DOI:** 10.1107/S1600536808021764

**Published:** 2008-07-31

**Authors:** Yong-Chuang Zhu, Dao-Hang He

**Affiliations:** a School of Chemistry and Chemical Engineering, South China University of Technology, Guangzhou 510640, People’s Republic of China

## Abstract

The title compound, C_17_H_17_BrN_2_O_4_·CH_4_O, was synthesized by the condensation of 3,4,5-trimethoxy­benzohydrazide and 2-bromo­benzaldehyde. The two aromatic rings are approximately planar, the dihedral angle being 3.08 (9)°. The mol­ecules are linked by inter­molecular N—H⋯O and O—H⋯O hydrogen bonds into chains along the *a* axis.

## Related literature

For related literature, see: Constable & Holmes (1987 ▶); Ganjali *et al.* (2006 ▶); Gardner *et al.* (1991 ▶); Jing *et al.* (2006 ▶); Kuriakose *et al.* (2007 ▶); Patole *et al.* (2003 ▶); Zhou *et al.* (2005 ▶).
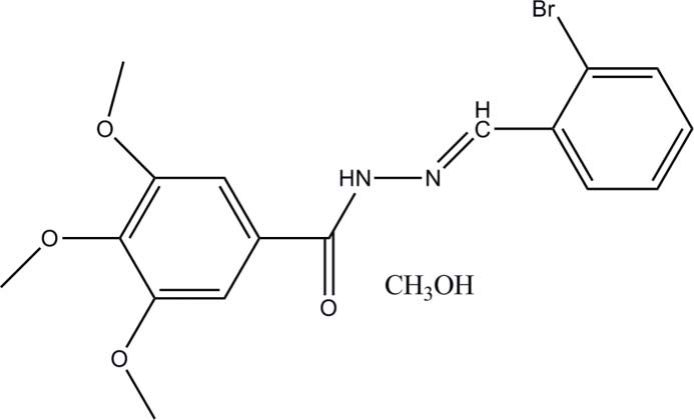


## Experimental

### 

#### Crystal data

C_17_H_17_BrN_2_O_4_·CH_4_O
*M* *_r_* = 425.28
Orthorhombic, 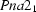

*a* = 12.9234 (7) Å
*b* = 4.9159 (3) Å
*c* = 29.3975 (17) Å
*V* = 1867.63 (19) Å^3^
*Z* = 4
Mo *K*α radiation
μ = 2.23 mm^−1^
*T* = 173 (2) K
0.36 × 0.35 × 0.33 mm

#### Data collection

Bruker SMART 1000 CCD diffractometer
Absorption correction: multi-scan (*SADABS*; Sheldrick, 2003 ▶) *T*_min_ = 0.455, *T*_max_ = 0.479
8158 measured reflections
3799 independent reflections
3206 reflections with *I* > 2σ(*I*)
*R*_int_ = 0.027

#### Refinement

*R*[*F* ^2^ > 2σ(*F* ^2^)] = 0.029
*wR*(*F* ^2^) = 0.080
*S* = 1.04
3799 reflections
240 parameters
1 restraint
H-atom parameters constrained
Δρ_max_ = 0.33 e Å^−3^
Δρ_min_ = −0.26 e Å^−3^
Absolute structure: Flack (1983 ▶), 1720 Friedel pairs
Flack parameter: −0.008 (8)

### 

Data collection: *SMART* (Bruker, 2001 ▶); cell refinement: *SAINT-Plus* (Bruker, 2003 ▶); data reduction: *SAINT-Plus*; program(s) used to solve structure: *SHELXTL* (Sheldrick, 2008 ▶); program(s) used to refine structure: *SHELXTL*; molecular graphics: *SHELXTL*; software used to prepare material for publication: *SHELXTL*.

## Supplementary Material

Crystal structure: contains datablocks I, global. DOI: 10.1107/S1600536808021764/wn2271sup1.cif

Structure factors: contains datablocks I. DOI: 10.1107/S1600536808021764/wn2271Isup2.hkl

Additional supplementary materials:  crystallographic information; 3D view; checkCIF report

## Figures and Tables

**Table 1:** Hydrogen-bond geometry (Å, °)

*D*—H⋯*A*	*D*—H	H⋯*A*	*D*⋯*A*	*D*—H⋯*A*
N1—H1*A*⋯O5^i^	0.88	2.01	2.871 (4)	164
O5—H5⋯O4	0.84	1.96	2.794 (3)	175

Symmetry code: (i) 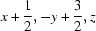.

**Fig. 1.**
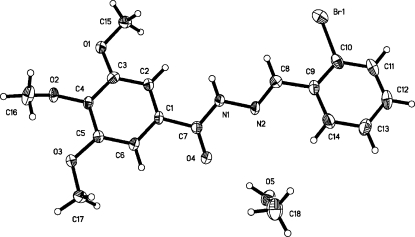
The structure of the two independent molecules in the asymmetric unit of the title compound, with the atom numbering. Displacement ellipsoids are drawn at the 50% probability level. Hydrogen atoms are represented by spheres of arbitrary radius.

**Fig. 2.**
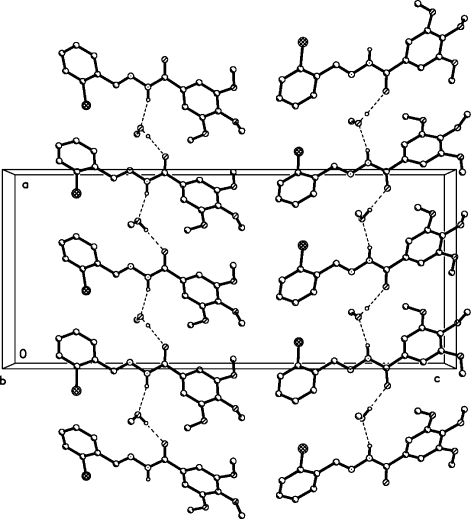
The packing of the title compound, viewed down the b axis. The dashed lines represent the hydrogen bonds. H atoms not involved in hydrogen bonding have been omitted.

## supplementary crystallographic information

### Comment

Hydrazones are acknowledged to possess a diverse range of bioactivities; these include antibacterial, antiviral, antineoplastic, and anti-inflammatory (Constable & Holmes, 1987; Ganjali *et al.*, 2006; Gardner *et al.*, 1991; Patole *et al.*, 2003). In addition, many hydrazones have also been used as ligands because they can readily form stable complexes with most metal ions (Kuriakose *et al.*, 2007; Zhou *et al.*, 2005). We report here the synthesis and crystal structure of the title compound, obtained by the condensation of 3,4,5-trimethoxybenzohydrazide and 2-bromobenzaldehyde.

The asymmetric unit of the title compound comprises one *N*'-(2-bromobenzylidene)-3,4,5-trimethoxybenzohydrazide and a methanol solvent molecule (Fig. 1). The two aromatic rings are approximately planar, with a dihedral angle of 3.08 (9)°. Similar geometry has been observed in related hydrazone analogues (Jing *et al.*, 2006). The methanol molecules in the crystal structure are linked to *N*'-(2-bromobenzylidene)-3,4,5-trimethoxybenzohydrazide through intermolecular N—H···O and O—H···O hydrogen bonds into chains along the *a* axis (Fig. 2).

### Experimental

A mixture of 3,4,5-trimethoxybenzohydrazide (1 mmol) and 2-bromobenzaldehyde (1 mmol) in anhydrous ethanol (10 ml) was refluxed for 2 h. When the solution was cooled to room temperature, some white needles separated out. After filtration, colorless single crystals suitable for X-ray analysis were obtained by slow evaporation of a methanol solution.

### Refinement

All H atoms were placed in geometrically idealized positions and allowed to ride on their parent atoms, with N—H = 0.88 Å, O—H = 0.84 Å, Csp^2^—H = 0.95 Å, C(methyl)—H = 0.98 Å and *U*_iso_(H) = *xU*_eq_(C, N, O), where *x* = 1.5 for the methyl and hydroxyl groups, *x* = 1.2 for all other H atoms.

### Crystal data

**Table:**

C_17_H_17_BrN_2_O_4_·CH_4_O	*F*_000_ = 872
*M**_r_* = 425.28	*D*_x_ = 1.512 Mg m^−^^3^
Orthorhombic, *P**n**a*2_1_	Mo *K*α radiation λ = 0.71073 Å
Hall symbol: P 2c -2n	Cell parameters from 4139 reflections
*a* = 12.9234 (7) Å	θ = 2.8–26.8º
*b* = 4.9159 (3) Å	µ = 2.23 mm^−^^1^
*c* = 29.3975 (17) Å	*T* = 173 (2) K
*V* = 1867.63 (19) Å^3^	Block, colorless
*Z* = 4	0.36 × 0.35 × 0.33 mm

### Data collection

**Table:**

Bruker SMART 1000 CCD diffractometer	3799 independent reflections
Radiation source: fine-focus sealed tube	3206 reflections with *I* > 2σ(*I*)
Monochromator: graphite	*R*_int_ = 0.027
*T* = 173(2) K	θ_max_ = 27.0º
ω scans	θ_min_ = 1.4º
Absorption correction: multi-scan(SADABS; Sheldrick, 2003)	*h* = −15→16
*T*_min_ = 0.455, *T*_max_ = 0.479	*k* = −2→6
8158 measured reflections	*l* = −34→37

### Refinement

**Table:**

Refinement on *F*^2^	Hydrogen site location: inferred from neighbouring sites
Least-squares matrix: full	H-atom parameters constrained
*R*[*F*^2^ > 2σ(*F*^2^)] = 0.030	*w* = 1/[σ^2^(*F*_o_^2^) + 0.8008*P*] where *P* = (*F*_o_^2^ + 2*F*_c_^2^)/3
*wR*(*F*^2^) = 0.080	(Δ/σ)_max_ = 0.001
*S* = 1.04	Δρ_max_ = 0.33 e Å^−^^3^
3799 reflections	Δρ_min_ = −0.26 e Å^−^^3^
240 parameters	Extinction correction: none
1 restraint	Absolute structure: Flack (1983), 1720 Friedel pairs
Primary atom site location: structure-invariant direct methods	Flack parameter: −0.008 (8)
Secondary atom site location: difference Fourier map	

### Special details

**Table:**

Geometry. All e.s.d.'s (except the e.s.d. in the dihedral angle between two l.s. planes) are estimated using the full covariance matrix. The cell e.s.d.'s are taken into account individually in the estimation of e.s.d.'s in distances, angles and torsion angles; correlations between e.s.d.'s in cell parameters are only used when they are defined by crystal symmetry. An approximate (isotropic) treatment of cell e.s.d.'s is used for estimating e.s.d.'s involving l.s. planes.
Refinement. Refinement of *F*^2^ against ALL reflections. The weighted *R*-factor *wR* and goodness of fit *S* are based on *F*^2^, conventional *R*-factors *R* are based on *F*, with *F* set to zero for negative *F*^2^. The threshold expression of *F*^2^ > σ(*F*^2^) is used only for calculating *R*-factors(gt) *etc*. and is not relevant to the choice of reflections for refinement. *R*-factors based on *F*^2^ are statistically about twice as large as those based on *F*, and *R*- factors based on ALL data will be even larger.

### Fractional atomic coordinates and isotropic or equivalent isotropic displacement parameters (Å^2^)

**Table:**

	*x*	*y*	*z*	*U*_iso_*/*U*_eq_	
Br1	0.61656 (2)	1.31840 (7)	0.659414 (16)	0.03707 (11)	
C1	0.5590 (2)	0.7908 (6)	0.89278 (10)	0.0193 (6)	
C2	0.6409 (2)	0.9755 (6)	0.89566 (11)	0.0195 (6)	
H2	0.6593	1.0831	0.8701	0.023*	
C3	0.6953 (2)	1.0008 (6)	0.93627 (11)	0.0207 (7)	
C4	0.6698 (2)	0.8389 (6)	0.97327 (10)	0.0186 (6)	
C5	0.5855 (2)	0.6608 (6)	0.97056 (11)	0.0215 (7)	
C6	0.5308 (2)	0.6357 (6)	0.93023 (11)	0.0211 (7)	
H6	0.4742	0.5129	0.9282	0.025*	
C7	0.4968 (2)	0.7557 (6)	0.85047 (11)	0.0212 (7)	
C8	0.5282 (3)	0.9221 (7)	0.73624 (12)	0.0276 (7)	
H8	0.5962	0.9952	0.7377	0.033*	
C9	0.4707 (3)	0.9235 (7)	0.69320 (11)	0.0254 (7)	
C10	0.4991 (2)	1.0835 (6)	0.65573 (14)	0.0264 (7)	
C11	0.4448 (3)	1.0814 (8)	0.61551 (12)	0.0337 (8)	
H11	0.4657	1.1955	0.5911	0.040*	
C12	0.3603 (3)	0.9139 (8)	0.61066 (13)	0.0362 (9)	
H12	0.3235	0.9084	0.5827	0.043*	
C13	0.3292 (3)	0.7520 (8)	0.64720 (12)	0.0344 (9)	
H13	0.2709	0.6357	0.6441	0.041*	
C14	0.3830 (3)	0.7604 (8)	0.68790 (14)	0.0312 (8)	
H14	0.3598	0.6530	0.7127	0.037*	
C15	0.8028 (3)	1.3547 (7)	0.90665 (12)	0.0254 (7)	
H15A	0.8276	1.2478	0.8807	0.038*	
H15B	0.8577	1.4779	0.9168	0.038*	
H15C	0.7422	1.4611	0.8975	0.038*	
C16	0.7819 (3)	0.6441 (7)	1.02794 (14)	0.0378 (9)	
H16A	0.7402	0.4792	1.0242	0.057*	
H16B	0.8003	0.6660	1.0601	0.057*	
H16C	0.8451	0.6289	1.0097	0.057*	
C17	0.4812 (3)	0.3298 (7)	1.00811 (13)	0.0294 (8)	
H17A	0.4160	0.4211	1.0006	0.044*	
H17B	0.4748	0.2407	1.0378	0.044*	
H17C	0.4968	0.1932	0.9848	0.044*	
C18	0.2740 (4)	0.2319 (9)	0.78016 (15)	0.0461 (11)	
H18A	0.3183	0.2281	0.7531	0.069*	
H18B	0.2092	0.1360	0.7737	0.069*	
H18C	0.3096	0.1426	0.8055	0.069*	
N2	0.4860 (2)	0.8218 (6)	0.77170 (9)	0.0247 (6)	
N1	0.5432 (2)	0.8335 (6)	0.81116 (9)	0.0254 (6)	
H1A	0.6079	0.8897	0.8109	0.030*	
O1	0.77516 (17)	1.1759 (4)	0.94291 (7)	0.0240 (5)	
O2	0.72395 (17)	0.8738 (5)	1.01327 (8)	0.0247 (5)	
O3	0.56273 (16)	0.5255 (5)	1.00977 (8)	0.0279 (5)	
O4	0.40936 (17)	0.6632 (5)	0.85149 (8)	0.0282 (5)	
O5	0.25214 (18)	0.5045 (5)	0.79189 (9)	0.0319 (6)	
H5	0.2973	0.5615	0.8101	0.048*	

### Atomic displacement parameters (Å^2^)

**Table:**

	*U*^11^	*U*^22^	*U*^33^	*U*^12^	*U*^13^	*U*^23^
Br1	0.03845 (18)	0.03817 (18)	0.03459 (19)	−0.00184 (16)	0.0079 (2)	0.0056 (2)
C1	0.0208 (15)	0.0234 (15)	0.0137 (16)	0.0046 (13)	−0.0037 (12)	−0.0005 (12)
C2	0.0212 (15)	0.0209 (15)	0.0165 (16)	0.0034 (13)	−0.0004 (12)	0.0008 (12)
C3	0.0181 (14)	0.0212 (16)	0.0228 (17)	0.0016 (13)	0.0009 (13)	−0.0054 (13)
C4	0.0209 (15)	0.0203 (15)	0.0144 (16)	0.0027 (13)	−0.0023 (12)	−0.0036 (12)
C5	0.0218 (15)	0.0231 (17)	0.0195 (17)	0.0003 (13)	−0.0024 (13)	0.0030 (13)
C6	0.0201 (15)	0.0238 (16)	0.0194 (17)	0.0007 (13)	−0.0051 (12)	0.0003 (13)
C7	0.0206 (15)	0.0247 (16)	0.0183 (16)	0.0010 (13)	−0.0012 (13)	−0.0004 (12)
C8	0.0235 (16)	0.0360 (18)	0.0232 (18)	−0.0025 (15)	−0.0004 (14)	0.0030 (15)
C9	0.0271 (17)	0.0318 (17)	0.0172 (17)	0.0048 (15)	−0.0029 (13)	−0.0024 (13)
C10	0.0313 (15)	0.0282 (14)	0.0196 (17)	0.0088 (12)	0.0059 (18)	−0.0007 (16)
C11	0.047 (2)	0.037 (2)	0.0170 (18)	0.0092 (18)	0.0041 (16)	0.0035 (15)
C12	0.047 (2)	0.043 (2)	0.0192 (19)	0.0102 (19)	−0.0100 (16)	−0.0003 (16)
C13	0.0344 (18)	0.042 (2)	0.027 (2)	−0.0003 (16)	−0.0079 (16)	−0.0059 (14)
C14	0.033 (2)	0.040 (2)	0.021 (2)	0.0007 (18)	−0.0008 (15)	0.0053 (14)
C15	0.0242 (16)	0.0260 (18)	0.0260 (19)	−0.0014 (15)	0.0021 (14)	−0.0016 (14)
C16	0.040 (2)	0.036 (2)	0.037 (2)	0.0072 (19)	−0.0187 (18)	−0.0032 (17)
C17	0.0250 (17)	0.0339 (19)	0.0294 (19)	−0.0035 (16)	0.0007 (14)	0.0083 (16)
C18	0.057 (3)	0.046 (2)	0.036 (2)	0.011 (2)	−0.004 (2)	−0.0082 (19)
N2	0.0217 (13)	0.0366 (16)	0.0159 (14)	−0.0002 (12)	−0.0058 (11)	0.0011 (12)
N1	0.0185 (13)	0.0413 (18)	0.0162 (14)	−0.0031 (13)	−0.0030 (10)	0.0024 (12)
O1	0.0251 (11)	0.0277 (12)	0.0193 (12)	−0.0046 (10)	−0.0042 (9)	0.0012 (9)
O2	0.0305 (12)	0.0285 (12)	0.0150 (12)	−0.0001 (10)	−0.0068 (10)	−0.0019 (9)
O3	0.0277 (12)	0.0379 (13)	0.0181 (12)	−0.0087 (11)	−0.0042 (10)	0.0067 (10)
O4	0.0232 (11)	0.0409 (14)	0.0205 (13)	−0.0073 (11)	−0.0049 (10)	0.0038 (10)
O5	0.0230 (12)	0.0402 (14)	0.0326 (14)	0.0039 (11)	−0.0045 (11)	−0.0067 (11)

### Geometric parameters (Å, °)

**Table:**

Br1—C10	1.911 (3)	C12—H12	0.9500
C1—C6	1.388 (4)	C13—C14	1.384 (5)
C1—C2	1.397 (4)	C13—H13	0.9500
C1—C7	1.491 (4)	C14—H14	0.9500
C2—C3	1.391 (4)	C15—O1	1.427 (4)
C2—H2	0.9500	C15—H15A	0.9800
C3—O1	1.358 (4)	C15—H15B	0.9800
C3—C4	1.388 (4)	C15—H15C	0.9800
C4—O2	1.379 (4)	C16—O2	1.422 (4)
C4—C5	1.400 (5)	C16—H16A	0.9800
C5—O3	1.363 (4)	C16—H16B	0.9800
C5—C6	1.386 (4)	C16—H16C	0.9800
C6—H6	0.9500	C17—O3	1.428 (4)
C7—O4	1.218 (4)	C17—H17A	0.9800
C7—N1	1.357 (4)	C17—H17B	0.9800
C8—N2	1.276 (4)	C17—H17C	0.9800
C8—C9	1.467 (4)	C18—O5	1.412 (5)
C8—H8	0.9500	C18—H18A	0.9800
C9—C14	1.398 (5)	C18—H18B	0.9800
C9—C10	1.402 (5)	C18—H18C	0.9800
C10—C11	1.375 (5)	N2—N1	1.377 (4)
C11—C12	1.375 (6)	N1—H1A	0.8800
C11—H11	0.9500	O5—H5	0.8400
C12—C13	1.396 (5)		
			
C6—C1—C2	120.5 (3)	C14—C13—H13	119.9
C6—C1—C7	117.1 (3)	C12—C13—H13	119.9
C2—C1—C7	122.3 (3)	C13—C14—C9	121.4 (4)
C3—C2—C1	119.5 (3)	C13—C14—H14	119.3
C3—C2—H2	120.2	C9—C14—H14	119.3
C1—C2—H2	120.2	O1—C15—H15A	109.5
O1—C3—C4	115.6 (3)	O1—C15—H15B	109.5
O1—C3—C2	124.3 (3)	H15A—C15—H15B	109.5
C4—C3—C2	120.1 (3)	O1—C15—H15C	109.5
O2—C4—C3	118.5 (3)	H15A—C15—H15C	109.5
O2—C4—C5	121.4 (3)	H15B—C15—H15C	109.5
C3—C4—C5	119.9 (3)	O2—C16—H16A	109.5
O3—C5—C6	124.7 (3)	O2—C16—H16B	109.5
O3—C5—C4	115.2 (3)	H16A—C16—H16B	109.5
C6—C5—C4	120.1 (3)	O2—C16—H16C	109.5
C5—C6—C1	119.7 (3)	H16A—C16—H16C	109.5
C5—C6—H6	120.2	H16B—C16—H16C	109.5
C1—C6—H6	120.2	O3—C17—H17A	109.5
O4—C7—N1	122.4 (3)	O3—C17—H17B	109.5
O4—C7—C1	121.5 (3)	H17A—C17—H17B	109.5
N1—C7—C1	116.1 (3)	O3—C17—H17C	109.5
N2—C8—C9	119.3 (3)	H17A—C17—H17C	109.5
N2—C8—H8	120.3	H17B—C17—H17C	109.5
C9—C8—H8	120.3	O5—C18—H18A	109.5
C14—C9—C10	116.5 (3)	O5—C18—H18B	109.5
C14—C9—C8	120.3 (3)	H18A—C18—H18B	109.5
C10—C9—C8	123.2 (3)	O5—C18—H18C	109.5
C11—C10—C9	122.6 (3)	H18A—C18—H18C	109.5
C11—C10—Br1	117.3 (3)	H18B—C18—H18C	109.5
C9—C10—Br1	120.1 (3)	C8—N2—N1	116.2 (3)
C12—C11—C10	119.9 (3)	C7—N1—N2	117.9 (3)
C12—C11—H11	120.0	C7—N1—H1A	121.1
C10—C11—H11	120.0	N2—N1—H1A	121.1
C11—C12—C13	119.4 (3)	C3—O1—C15	118.2 (2)
C11—C12—H12	120.3	C4—O2—C16	115.3 (2)
C13—C12—H12	120.3	C5—O3—C17	117.4 (3)
C14—C13—C12	120.2 (4)	C18—O5—H5	109.5
			
C6—C1—C2—C3	0.9 (4)	C14—C9—C10—C11	−0.5 (5)
C7—C1—C2—C3	179.4 (3)	C8—C9—C10—C11	179.6 (3)
C1—C2—C3—O1	−179.2 (3)	C14—C9—C10—Br1	179.1 (2)
C1—C2—C3—C4	1.5 (4)	C8—C9—C10—Br1	−0.9 (4)
O1—C3—C4—O2	2.0 (4)	C9—C10—C11—C12	−1.2 (5)
C2—C3—C4—O2	−178.6 (3)	Br1—C10—C11—C12	179.2 (3)
O1—C3—C4—C5	177.0 (3)	C10—C11—C12—C13	1.5 (5)
C2—C3—C4—C5	−3.6 (4)	C11—C12—C13—C14	0.0 (6)
O2—C4—C5—O3	−0.7 (4)	C12—C13—C14—C9	−1.8 (6)
C3—C4—C5—O3	−175.6 (3)	C10—C9—C14—C13	1.9 (5)
O2—C4—C5—C6	178.2 (3)	C8—C9—C14—C13	−178.1 (3)
C3—C4—C5—C6	3.3 (4)	C9—C8—N2—N1	−178.4 (3)
O3—C5—C6—C1	177.8 (3)	O4—C7—N1—N2	4.1 (5)
C4—C5—C6—C1	−1.0 (5)	C1—C7—N1—N2	−175.7 (3)
C2—C1—C6—C5	−1.2 (5)	C8—N2—N1—C7	173.1 (3)
C7—C1—C6—C5	−179.7 (3)	C4—C3—O1—C15	−177.9 (3)
C6—C1—C7—O4	21.3 (4)	C2—C3—O1—C15	2.7 (4)
C2—C1—C7—O4	−157.2 (3)	C3—C4—O2—C16	−117.3 (3)
C6—C1—C7—N1	−159.0 (3)	C5—C4—O2—C16	67.8 (4)
C2—C1—C7—N1	22.5 (4)	C6—C5—O3—C17	4.7 (5)
N2—C8—C9—C14	−15.7 (5)	C4—C5—O3—C17	−176.5 (3)
N2—C8—C9—C10	164.3 (3)		

### Hydrogen-bond geometry (Å, °)

**Table:**

*D*—H···*A*	*D*—H	H···*A*	*D*···*A*	*D*—H···*A*
N1—H1A···O5^i^	0.88	2.01	2.871 (4)	164
O5—H5···O4	0.84	1.96	2.794 (3)	175

Symmetry codes: (i) *x*+1/2, −*y*+3/2, *z*.
